# The United States Dissector, or Lessons in Practical Anatomy

**Published:** 1846-10

**Authors:** 


### PART III.—BIBLIOGRAPHICAL NOTICES.

### ARTICLE V.

*The United States Dissector, or Lessons in Practical Anatomy.* By WM. E. HORNER, M. D., Prof. of Anatomy in the University of Pennsylvania. Fourth addition, with numerous illustrations. Edited by HENRY H. SMITH, M. D., Fellow of the College of Physicians, of Philadelphia, &c. Philadelphia: Lea & Blanchard. 1846. pp. 444. (For sale by Brautigam & Keen, Chicago.)

This is a reprint of "Horner's Practical Anatomy," a work which has for more than twenty years been before the profession, and almost the only one of the same character by an American author. It is a work of established reputation, and the illustrations added to this addition are well chosen, so that it will compare favorably with the best "Dissectors."

